# Policy Innovation and Policy Pathways: Tuberculosis Control in Sri Lanka, 1948–1990

**DOI:** 10.1017/mdh.2016.58

**Published:** 2016-10

**Authors:** Margaret Jones

**Affiliations:** Research Fellow and Deputy Director, Centre for Global Health Histories, Department of History, University of York, York YO10 5DD, UK

**Keywords:** Sri Lanka, UNICEF, Tuberculosis, WHO, Community, Integration

## Abstract

This paper, based on World Health Organization and Sri Lankan sources, examines the attempts to control tuberculosis in Sri Lanka from independence in 1948. It focuses particularly on the attempt in 1966 to implement a World Health Organization model of community-orientated tuberculosis control that sought to establish a horizontally structured programme through the integration of control into the general health services. The objective was to create a cost- effective method of control that relied on a simple bacteriological test for case finding and for treatment at the nearest health facility that would take case detection and treatment to the rural periphery where specialist services were lacking. In the late 1940s and early 1950s, Sri Lanka had already established a specialist control programme composed of chest clinics, mass X-ray, inpatient and domiciliary treatment, and social assistance for sufferers. This programme had both reduced mortality and enhanced awareness of the disease. This paper exposes the obstacles presented in trying to impose the World Health Organization’s internationally devised model onto the existing structure of tuberculosis control already operating in Sri Lanka. One significant hindrance to the WHO approach was lack of resources but, equally important, was the existing medical culture that militated against its acceptance.

## 1 Introduction

The Eighth Report of the World Health Organization’s (WHO) Expert Committee on Tuberculosis in 1964 recommended that ‘a national tuberculosis programme must be on a country-wide and permanent basis’ and that ‘tuberculosis services [should] be integrated into general health services’.^1^ The blueprint for tuberculosis control outlined in this report represented an attempt to grapple with what the then Director General of the WHO, Dr M.G. Candau, described as a ‘public health problem of major importance in all countries’ made even more pressing by ‘the disturbing fact of a rapidly increasing gap... between the economic “have” and the “have not” countries’.^2^ This growing divergence between rich and poor nations in their epidemiological experience of tuberculosis in the decades following the Second World War highlighted the need to find a method of control that was feasible in areas with scarce resources, facilities and trained personnel.^3^ The advent of effective chemotherapies from the 1950s that appeared to promise a new environment for disease control suggested, according to this Expert Committee, that the ‘unsatisfactory position in tuberculosis was to a large extent due to the inadequate application of existing knowledge’.^4^ This conclusion illustrates the optimistic assumption of the period that the technological expertise and therapeutic knowledge of Western medicine could finally rid the world of the worst scourges of disease without tackling the underlying socio-economic conditions.^5^

Early hopes for an effective cure for tuberculosis induced by the introduction of streptomycin in 1946 were soon dashed due to the emergence of resistance to the drug. Further clinical trials established that combining streptomycin with para-aminosalicyclic (PAS) and isoniazid (INH) dealt with the resistance problem but, as these drugs were expensive and required, it was thought, a year’s stay in a hospital, it was a regimen that could only be used in developed countries.^6^ It was not a model that could be universalised throughout the world, but further breakthroughs established parameters for control that could serve the objectives of international health policy makers after the Second World War. The 1956–59 Wallace Fox trials at the Madras Tuberculosis Chemotherapy Centre showed that domiciliary chemotherapy could be as effective as expensive drug treatment in a hospital or sanatorium.^7^ However, at the same time, the trials highlighted the problem of ensuring that outside of trial conditions the drugs were taken regularly and that the course was completed. As Sunil Amrith has argued, the Madras study had ‘ample resources’, and could and did ‘exercise a particularly high level of social control over the daily lives of the patients in the study’ and the patients were selected on the basis that they would be ‘cooperative’; circumstances hardly commensurate with practical realities.^8^ Further trials at the Madras Centre, East Africa, Hong Kong and Singapore in the 1960s produced a partial solution; a six-month, combination, intermittent drug regime whereby patients attended clinics twice a week for chemotherapy appeared to ease the problem of patient supervision. This short course therapy ultimately took shape in the WHO DOTS strategy of the 1990s. DOT stands for Directly Observed Therapy. Patients took their medication in the presence of medical staff.^9^

The community model of tuberculosis control advocated in the 1964 report therefore derived directly from the Madras and subsequent trials and formed the basis of India’s National Tuberculosis Programme, established in 1961.^10^ This paper analyses the attempt to transfer this WHO/Indian model to India’s southern neighbour, Sri Lanka between the years 1966 and 1972. As such, it explores, in a very different social, political and medical context, issues raised by existing studies of tuberculosis control in developing countries and contributes to an understanding of the nature and significance of international public health in both policy and praxis in the post-colonial world.^11^ Sri Lanka was not India; it had, by the mid-1960s, an extensive health care infrastructure, low mortality rates, high life expectancy and literacy rates for a low-income country.^12^ Moreover, by 1966, it already had a specialist tuberculosis control programme (TCP) and a high degree of public awareness of the disease. Exploring how the community approach was transferred to this particular environment militates against the tendency of some historians, as Lauren Minsky has highlighted, of ‘aggregating’ evidence from some locales in order to produce a ‘global linear narrative’.^13^ Sri Lanka, for example, does not fit easily into a South Asian model of internationally influenced public health that is rooted in the experience of India.^14^ Historians, as well as policy makers, have a duty to recognise differences in historical trajectories and cultural contexts and not, to borrow Christian McMillen’s phrase, ‘flatten the world into an undifferentiated mass’.^15^

This paper broadly divides into three sections. The first section briefly explores the development of the specialist TCP referred to above. The second focuses on the policies and mechanisms by which the community-orientated WHO policy was to be implemented and the last section explores how and why it ultimately failed to reach its policy objective.

## 2 Tuberculosis and Tuberculosis Control in Sri Lanka, 1910–66 – a Summary

The colonial government of Sri Lanka had identified tuberculosis as a major health threat from the beginning of the twentieth century. Annual death rates, estimated to be about 4000 between 1905 and 1910,^16^ led to the formation of a Government Committee in 1910 to examine its causes and consider responses.^17^ As a result, the first specialist services were set up: an Anti-Tuberculosis Institute and dispensary in Colombo in 1916, a hospital for advanced cases at Ragama in 1917, the Kandama Sanatorium became fully operational in 1919 and a second sanatorium was established at Kankesanturai in 1931. Chest clinics in Colombo and Jaffna also followed. Tuberculosis was made a notifiable disease, although this was not enforced.

Nevertheless, in 1948, the Director of Medical Services (DMS) acknowledged that ‘with the control of Malaria’, tuberculosis had ‘become the most serious medical and socio-economic problem in Ceylon’.^18^ A sample survey of the population of Kotte in 1944, a small urban area to the south of Colombo, had revealed a tuberculosis morbidity rate of 2.2 per cent and a death rate of 470 per 100 000 while, according to the official statistics on tuberculosis, the death rate in Kotte was only 60–80 per 100 000. Although these figures could not be directly extrapolated to the whole island, they did suggest, Dr W.G. Wickremesinghe (DMS) argued, that the actual death rate from tuberculosis was much higher than the official figure for 1948 of 57.5 per 100 000. He concluded that there were likely to be more than 100 000 cases of ‘open’ tuberculosis (ie. actively infectious) in a population of about seven million.^19^ The tuberculosis clinics and hospitals had focused primarily on treatment, but the realisation of the scale of the problem shifted attention onto detection and prevention. A first batch of medical officers had already been sent for specialist training in tuberculosis work to India in 1942 and the Kotte survey gave rise to a second Government Committee on Tuberculosis in 1943.

This second Tuberculosis Committee resulted in the establishment of an extended vertical control programme based on a model of control devised in the developed world. A Superintendent of the Anti-Tuberculosis Campaign for the coordination, control and development of anti-tuberculosis work was appointed in 1945 alongside a staff of seven medical officers (MOs), seven assistant MOs, nurses, X-ray and laboratory workers and a sanitary inspector.^20^ The number of MOs annually receiving specialist TB training in India was increased from three and, in 1954, a school for training TB nurses was opened at the Welisara Chest Clinic.^21^ Treatment of the disease involved both institutional and domiciliary care. It was accepted that more beds were needed for inpatient care in the specialist hospitals and clinics and that separate wards were needed in the general hospitals. In areas where there were no beds, notably in rural areas, domiciliary care was ideally provided to patients waiting for a bed, to discharged patients or to those who were unwilling or unable to enter a hospital.^22^ The treatment, until the introduction of streptomycin in 1951, focused on the standard care of isolation (in homes or institutions), rest, good food and collapse therapy.^23^ As a result of the TB expert Donald Barlow’s report in 1952, which highlighted the deficiencies of the system, a grant of Rs 3 million was negotiated through the Colombo Plan from the Australian government for the establishment of chest clinics in every province.^24^

Treatment was only one aspect of control; the other two were case detection and prevention. Sufferers, even if they were aware of what their symptoms signified, did not always present themselves for treatment as confirmation of the disease resulted in loss of employment, destitution and social stigma (hence the discrepancy between the official statistics and actual cases of the disease, as thrown up in the Kotte survey, for example).^25^ Before the advent of modern chemotherapy, there was little point in incurring these risks as the disease could only rarely be cured even with the best of care. Public health education was an essential tool for changing this pattern of behaviour and, to this end, the Ceylon National Association for the Prevention of Tuberculosis (CNAPT) was established in 1949 by Dr J.H.F. Jayasuriya with the support of the government and medical department. Again, this followed the model of similar associations in the West. The objectives of this association were the education of the general public, the creation and maintenance of interest in tuberculosis matters, the collection of funds for tuberculosis work and participation in the care and aftercare of patients.^26^ The lynchpin of preventive strategy was a BCG vaccination campaign, which began in 1949 with the help of the International Tuberculosis Campaign and UNICEF. Initially aimed at school children and selected adult groups in Colombo,^27^ after 1951, it was extended throughout the island and, by the mid- 1960s, it was offered to all babies born in government institutions and to school children.^28^ A planned case-finding campaign with mass miniature radiography was instigated in 1951.^29^ In 1953, to encourage patients to present themselves for treatment, a social assistance scheme for patients and families was set up by the government and, in the same year, combined Streptomycin and Isoniazid chemotherapy was introduced for all patients.^30^ Along with the Central Register of all known tuberculosis cases, which was started in 1958, these developments laid down the structure of the national TCP.

The first decade of the TCP was lauded as a success. The DMS claimed, in 1956, that tuberculosis sufferers had a ‘full range of medical treatment available, without delay at the special institutions and the usual hospitals’.^31^ There were six – soon to be nine – chest clinics, over 3500 tuberculosis beds for inpatients, supplemented by ambulatory or domiciliary care, financial assistance for sufferers and protection available for families and friends through the BCG vaccination.^32^ Sir Bennett Hance (Medical Adviser to the British Department of Commonwealth Relations) in his 1956 report for the Sri Lankan government on the health services concluded that: ‘Progress despite the limited staff has been spectacular’; evidenced by the halving of death rates from 528 per million of population in 1949 to 210 in 1954, which he attributed to ‘increased effective treatment’.^33^ Furthermore, the increasing morbidity rate was ‘indicative not of a seriously increasing problem but of better and earlier diagnosis with greatly improved chances of cure’.^34^ While he noted that assistance had been received from the WHO and UNICEF (especially for the BCG campaign), this success was, he considered,

wholly due to the Ministry and Department of Health, and to these belong the credit. Inspired by leadership and enthusiasm at the top, the workers in the campaign have toiled early and late against almost every conceivable handicap with a devotion which is worthy of the highest praise.^35^

If support from the departments of housing and agriculture for dealing with the problems of bad housing and poor nutrition was forthcoming, it would not be ‘unduly optimistic to say that *if present progress and momentum be maintained*, the disease should come under control (and here elimination is *not*implied) in a period of 10–15 years’ (italics in original).^36^

Not all agreed with Hance’s rather sanguine report. A sample survey conducted by Dr James Deeny from the WHO, on behalf of the government, led him to estimate that there were nearly 69 000 ‘unhealed’ cases, aged ten or over, that is, about one per cent of the population, and nearly 35 000 ‘healed’ cases. Significantly, 81.7 per cent of those unhealed cases in Deeny’s sample were found among the rural population. Since this group represented about 83 per cent of the total population, it suggested that the prevailing TCP was failing to reach a large proportion of people.^37^ In 1964, it was further estimated that there were nearer 80 000 cases, but only half of these had been diagnosed and that the majority of patients who did appear in the Central Register of Tuberculosis came from urban or semi-urban areas.^38^ As an illustration of the regional differences in the spread of TB services, a project to initiate supervised bi-weekly treatment in the Jaffna region (mainly Tamil) in 1965 was described by the CNAPT as the ‘first and concerted joint effort to arrest the spread of Tuberculosis in Jaffna’.^39^ It was such inequality of access to TB services that the WHO community approach was specifically designed to alleviate. The best way of ‘economically and efficiently’ rectifying the situation, according to the UNICEF/WHO plan for Sri Lanka, devised at the end of 1965, was ‘by integrating tuberculosis control into the existing and developing general health services’. This would have two advantages. Firstly, it would take ‘case finding activities out to the periphery on a permanent basis’ and, secondly, deal with the ‘defaulter’ problem by enabling ‘close supervision’ of detected cases to ensure regular and complete treatment.^40^ However, what this plan overlooked was that there was also an urban/semi-urban bias in the distribution of the general health agencies as they, like the specialist services, were concentrated in the more populous south and west of the island. Moreover, the rural peripheral health agencies were poorly resourced in comparison to their urban counterparts and contributed to the well-established behaviour pattern of Sri Lankans of by-passing their nearest health agency as inadequate and seeking medical aid from the central clinics and hospitals.^41^

The new WHO community project was the joint responsibility of the WHO, which provided the technical advisers, UNICEF, which provided supplies and equipment to the value of $42 000, and the government, which committed itself to providing all other personnel, supplies and equipment.^42^ It was structured to run parallel with the existing fifteen health divisions of the general health care services, with a divisional tuberculosis officer to head each unit. The existing TCP and its personnel remained in place but it was expected that, in time, the new model would graft naturally onto the old and the two would become integrated. Success for the WHO programme lay primarily in the number of infectious cases detected and successfully treated, and hence the importance of effective case finding. Under the WHO methodology, technology was uppermost and case detection was a simple matter; a ‘case’ was defined as a ‘person suffering from bacteriologically confirmed disease’ through the testing of the patient’s sputum and all a health worker needed for diagnosis therefore, apart from the patient’s cooperation, was “a microscope, basic technical knowledge and clear instructions”.^43^ The problem of incomplete chemotherapy which led both to the failure of treatment and the development of drug resistance was mitigated by organising treatment at the nearest general health facility to the patient, whether it be a dispensary, a health unit or out-patients department, and not just at the specialist chest clinics as hitherto. This would make treatment more accessible to all, especially in those areas far from specialist services and make it easier for health authorities to follow up patients and their contacts.^44^ In addition, the BCG vaccination programme was to be extended to cover as many babies, infants and school children as possible through maternity hospitals, welfare clinics and schools and with the introduction of just one injection of a freeze dried vaccine.^45^

The WHO recognised that the implementation of this method should take account of different contexts and that, in order to assess whether the plan was suitable for local conditions, a test area should be selected that was representative of socio-economic conditions and which, in time, could evolve into a demonstration and training area.^46^ The area chosen for this in Sri Lanka was the North Western Province (NWP), an area of 4826 square km, with 4958 villages and an estimated population of 1157 082. It was divided into two administrative districts centred on the towns of Kurunegala and Puttalam with nine health areas. Crucially, the NWP had a ‘very good coverage of basic health service units’ which, for the most part, were ‘accessible by transport services’ and it had a ‘comprehensive postal service’.^47^ This last factor was essential given the case-detecting method that relied upon sending samples to laboratories and receiving the results within a two-week time span. The province had some urban areas although it was largely rural and thus it could claim to broadly follow the national profile.

The WHO archives hold many of the quarterly reports jointly compiled by the WHO project leader and the Sri Lankan head of the programme, plus reports from outside experts who were sent to evaluate the project’s progress. Frequently they provided, as Amrith has highlighted, an ‘example of the ways in which technical assistance could contain a critique of itself’.^48^ These reports provide ample evidence that the community approach was not working in Sri Lanka but, as McMillen has shown in his recent study, in their rush to control TB (undoubtedly a laudable aim) the WHO had a tendency to brush aside problems as they arose in the field and plough on regardless.^49^ A distinctly Sri Lankan perspective is also sought within the pages of the newsletters of the CNAPT. These sources expose the deviations, negotiations and resistances that emerged at the national, local and individual level in the implementation of this internationally conceived policy.

## 3 The North Western Province Pilot Project

The pilot project began in the NWP in May 1966 under the joint direction of Dr J.V. Seneviratne (Sri Lankan and formerly Director of the Welisara Chest Clinic) and the WHO’s Dr F.J. Loven (later replaced by Dr Eung Soo Han).^50^ In addition, there was a laboratory technician, a public health inspector and educator, and a public health nurse. These subordinate staff were all Sri Lankan and had received specialist training via WHO fellowships at the National Tuberculosis Institute, Bangalore, India.^51^ In the two health divisions of the NWP, Kurunegala and Puttalam, there were ten Medical Officers of Health (MOH) areas, which were further sub-divided into 112 ‘ranges’ under a Public Health Inspector (PHI). All MOHs and PHIs were to participate fully in the integrated programme and all hospitals, bases, districts, peripherals and dispensaries to which a permanent MO was attached, were designated ‘centres’ for sample collection. It was through these centres that the objective of developing ‘permanent facilities’ to detect cases of tuberculosis among ‘symptom-motivated persons’ and organising a ‘satisfactorily functioning treatment services’ was to be implemented.^52^

Ninety-two of the general health agencies were designated as sputum collection centres in the NWP.^53^ A sputum sample was collected from all patients who presented at these centres suffering from a cough of more than two weeks duration or patients over the age of thirty-five who had shorter cough duration but who also had a fever and/or other chest symptoms. If a first specimen was negative but symptoms persisted, then a second sample was collected and, if this was negative and symptoms still remained, then the patient was referred on for chest X-ray to the established chest clinics at Kurunegala, Puttalam, Chilaw and Narawila.^54^ Under the existing programme, the chest X-ray and clinical assessment operated as the diagnostic methods of choice. Each collecting centre was supplied with slides, waxed ice cream cartons for the sputum samples, a wooden spoon, grease pencil, spirit lamp, boxes for transporting the slides and the forms for recording cases. Slides were sent by post weekly for laboratory examination and laboratory staff were requested to send the results back to the centres within five days.^55^ The attractions of this method of case finding to its Sri Lankan and WHO adherents are obvious. If it all worked smoothly, then it was cheap, as there was no costly technology involved; it utilised existing services so it entailed no new substantial outlay on facilities; and, when extended throughout the whole island, the hope was that it could reach those patients in rural areas who had been unable to access the existing specialist tuberculosis clinics but who could get to their nearest health facility.

Patients who tested positive were to be immediately offered treatment at the centre closest to their homes and which had been supplied with the appropriate drugs by the project MO, funded by the government and free to the patient. Standard treatment consisted of twice weekly injections of 1g of Streptomycin plus 700g of INH (Isoniazid), given at the same time as the injection, and 5–6g of pyridoxine, for one year. Sputum samples were supposed to be tested again after six months and at the end of one year, at which point those patients who still tested positive were referred to the specialist clinics, as they were assumed to be drug resistant and were considered for second-line drugs. This method of giving the drugs, administered under supervision and close to home, was thought to ensure that the treatment regime would be completed without the expense and difficulties of domiciliary visits.^56^ Furthermore, the new bi-weekly drug regime was a considerable improvement on the previous one that had entailed patients being sent home with the task of having to take thirty or more pills daily and unsupervised for several months. Understandably, as H. Goonewardene (CNAPT Secretary) acknowledged, patients found this ‘irksome’, and in ‘some cases nauseating’ and ‘one can quite understand how very difficult the patient finds it to continue regularly his self-administration of drugs’.^57^ However, even under the new regime, the problem of patients who did not turn up for their weekly treatment – the so-called defaulters – remained. These patients were the responsibility of the range PHI who was expected to visit the local centre on a weekly basis to pick up their names, trace them and ‘remotivate’ them.^58^ The sources contain no figures on the extent of patients who failed to complete treatment; the incentive for them to do so was that regular attendance for treatment was a condition for government social assistance. This, however, could work both ways; it was claimed that some patients preferred to remain ill and not complete treatments in order to retain their right to the government allowances.^59^ Reasons cited by CNAPT workers for interruptions or cessation of treatments included changes of address (so patients disappeared from the system), absences from home for business, work or pilgrimage (for Muslims, Ramadan created difficulties) and there was also outright refusal. For example, the stigma of tuberculosis in the family made sons and daughters unmarriageable so families did not want their members to attend clinics and be identified as sufferers.^60^

The long-term objective was for the NWP pilot project to become a training centre for all national health personnel in tuberculosis control methods, preparatory to the expansion of the integrated control programme throughout the whole island. Once the programme was up and running effectively in the NWP, then it could become the focal point for training health workers throughout the island, so it was essential to ensure that the initial problems of the programme were identified and solved. In its first eighteen months, the pilot project received some praise from WHO advisers. Dr K.L. Hitze, WHO Regional Adviser, considered that it had made a ‘remarkable start’ and that there was ‘every possibility that this provincial TB Control Programme may become the most advanced and integrated TB Programme in this region’.^61^ N. Cobbald, WHO Laboratory Technician, also noted that a ‘great deal of work and effort have been put into the integration of the health agencies of the NWP by the medical officer and the staff of the pilot project’.^62^ By the end of 1969, it was proposed to extend the programme to the Western Province in 1970, Sabaragamuwa and Eastern Provinces in 1971, Northern, Southern and Uva Provinces in 1972 and the whole island by 1973.^63^ Testament to this perceived success was a WHO seminar held in Colombo on 22–28 February 1970 which was attended by participants from Burma, Nepal and Thailand as well as fifty-one Sri Lankan participants and observers. They represented tuberculosis control officers, public health administrators, professors of medicine and public health, epidemiologists and statisticians.^64^ It was noted at the seminar that, in the NWP pilot project, the peripheral agencies had detected about one third of positive cases and, significantly, these cases came from a different age cohort than those detected at the provincial headquarters. They were people who lived away from the main roads and thus ‘would have had little chance of being detected by the specialized services’.^65^ It was precisely this group of patients that the integrated programme was designed to reach. The seminar participants endorsed the programme and proposed that the NWP should become an international training centre for an integrated tuberculosis service.^66^

However, despite these declarations of success, it was clear from the outset that the design of the project presented problems; some related to what could be expected in any attempt to implement a model devised at international level but aimed at a comparatively under-resourced country. Others, however, related specifically to the Sri Lankan context; in effect, the established health-seeking behaviour of Sri Lankans and the presence of an existing and functioning TCP exerted their own pull.

## 4 Problems of Resources

UNICEF supplied the necessary specialist equipment; this included microscopes, cameras, X-ray films, sputum containers, records cards and vehicles.^67^ These supplies did not always arrive on time or were not always suitable for the specific conditions of the island. For example, two vehicles were supplied to the project so that its leaders could travel to the health agencies and provide the necessary supervision and training of staff. Dr Hitze, the WHO adviser, on his visit in October 1966, had personally experienced the inadequacy of the two Volkswagon vehicles supplied by UNICEF; ‘repeatedly, because of the low road clearance, the bottom of the car hit the stony surface of the ‘road’.^68^ It was essential, he argued, that Land Rovers with four-wheel drive were provided so that all roads could be negotiated at any time ‘as is required for the job of permanent supervision’. He also asked for spare tyres for the existing VW cars because they needed frequent changing given the condition of the roads.^69^ Evidently, this plea was not responded to with any haste: in the first quarter of 1968, the project had been held up – in particular, the field visits – because one of the VWs had been in the garage for a month as there were no spare parts for repair.^70^ By 1969, some action had been taken; Dr Han reported in June 1969 that Land Rovers had been supplied by UNICEF but that they had not yet been delivered to the project.^71^ Delays also occurred in the delivery of the Land Rovers when the project was extended to the CP (it took three months there) and the NCP.^72^ Delays in the delivery of UNICEF supplies was apparently a constant feature of the programme but Dr Han’s recommendation in June 1969 that ‘UNICEF supplies be more expeditiously handled and delivered to projects concerned’ suggests that the fault may have been as much with the government’s organisational capacity as with UNICEF’s.^73^

The crucial component of detecting cases through sputum collection and testing by the general health agencies threw up many problems in relation to equipment, technical expertise and facilities. The task of collecting a viable sample depended upon having a suitable receptacle for its collection, an effective means of taking slides, a secure system for the packaging and posting of slides, equipped laboratories and trained staff. Additionally, there was the question of how the sample was to be collected from the patient. All these steps in the process had to be done properly to ensure the viability of the specimens and the accuracy of the testing and it is apparent that these procedures constantly failed to come up to the expected standards for various reasons. The sputum cups provided by UNICEF at the start had screw lids and could be sterilised and reused but Dr Hitze advised on his visit that these should only be used when the sputum sample was cultured at the Kurunegala provincial laboratory (cultures were advocated when a positive diagnosis could not immediately be identified from the sample). Instead, locally purchased ice cream tubs (at four cents each) were used.^74^ The ice cream industry also supplied the wooden spoons that staff were expected to use to select the portions of sputum for putting onto slides. Complaints that they were too short and exacerbated the difficulty and distastefulness of the process were upheld by N. Cobbald who recommended the use of locally sourced coconut leaf fibre sticks cut to six inch lengths.^75^

It was deemed that to get a good specimen of sputum, samples should be collected from patients under staff supervision. To N. Cobbald this should have presented no problems: ‘Even though the supervised collection does take more time (approximately three to five minutes), the time factor cannot be considered decisive when quality is essential.’ Ideally, sample collection could be done by any member of the centre staff but he had to concede that this had proved problematic: ‘Supervised sputum collection was not particularly well received, mainly because of the time factor and the “obnoxious” nature of the task.’ This had meant that often in smaller centres it was the MO who had to do it as the dispenser or labourer ‘could not be persuaded to undertake this task’.^76^ No acknowledgement was made of the already burdensome workload of the local MO who had to deal with patients presenting across the full range of the medical spectrum.^77^ Taking some account of the different circumstances he encountered, Cobbald suggested that, whenever possible, ‘sputa should be collected in the open air and, so as not to cause embarrassment to the patient, out of the sight of others’ and that ‘collection in crowded, non-ventilated rooms should be avoided’. He also advised that patients who had been ‘chewing betel or eating immediately before the collection’ should rinse their mouths with clean water before collection.^78^ On the face of it this last injunction appears a simple piece of practical advice to follow. However, it depended upon the availability of clean water in the unit and, in many health agencies, as Han and Richards noted in their field report of March 1969, there were often no such facilities. This lack was even more important when it came to supervised treatment. Patients could not take pills at the health unit in front of staff if there was no water for swallowing them. Han and Richards suggested that perhaps the local branches of the CNAPT could be ‘encouraged to provide utensils for storing drinking water and cups for the patients’.^79^ As noted before, the rural peripheral health agencies, the Cinderellas of Sri Lanka’s health care infrastructure, were inadequately resourced. These problems related to them particularly; the established chest clinics were much better equipped to fulfil these tasks.

The same kinds of deficiencies are also evident in the process of case detection. Reliance on the bacteriological test depended upon the availability of well-equipped laboratories and sufficient suitably trained staff, Cobbald’s visit in August 1967 revealed that, even with the supplies from UNICEF and support from the government and medical authorities, these could not just be conjured up. One major problem lay with the preparation of the slides at the general health agencies. Cobbald noted that smear preparation at the centres was inadequate; most of the smears were too thin. His detailed instructions to staff at the centres he visited are indicative of the deficiencies he perceived. He stressed the need to collect thick smears that would be most likely to contain the TB bacilli, which should cover two thirds of the available space on the slide, and he laid down guidelines for the ‘cleanliness of the slide’, for its labelling, for drying the smear and for its fixation and packing for postal transportation. He also suggested that the provision of a standard tin box container for transporting the smears from the centre to the laboratory (instead of the wide variety of round tins in use) would save time on the packing.^80^ The practical difficulties highlighted here illustrate the imperative of exploring what Michael Worboys has described as the ‘performative aspects of the germ practices’ as well as their ‘rationales and meanings’.^81^ A year later, in 1968, Dr C. Baily, another WHO adviser, noted that adequate preparation of the slides was still presenting difficulties; in particular, the fixing of the slides which ‘sometimes arrived at the reference laboratory with the wrapping paper adhering to them’. Baily’s solution was that wherever a microscope was available, then the specimen could be examined at the health agency where it was taken.^82^ He stressed that ‘continual control by the laboratory supervisor of the sputum case-finding procedures... especially, the quality control of sputum collection and smear preparation at the peripheral centres, is most essential’ but to rely even more on the peripheral agency staff for testing samples was merely glossing over the resource problem (underlining in original).^83^ Moreover, working microscopes were not always available even in the laboratories where they were obviously the essential piece of equipment.

Cobbald extended some praise to the six laboratories involved in the project in August 1966; cooperation was good and the staff ‘were found fully competent to carry out the necessary examinations’. He suggested changes in practice that were, he claimed, ‘fully acceptable to the staff’. Before he left, he instituted a quality control assessment procedure for training and supervision purposes.^84^ However, he also highlighted the problems with the equipment and put in a plea, in particular, for more microscopes. For example, Kuliaputiya laboratory had two technicians but only one microscope and they, of course, had more than just the TB slides to deal with. The Puttalam District Hospital Laboratory microscope was in a ‘poor state of repair’ so that ‘any reliable microscopy’ was ‘practically impossible’.^85^ The laboratory staff’s role was also expected to move beyond the purely technical as they were enjoined by Cobbald to be ‘more active in motivating centres to pay more attention to the quality of specimens and smears’.^86^ He ‘strongly suggested that the medical laboratory technician’ make at least twice-monthly field visits to participating laboratories to ensure they followed correct procedures and visit any health centres ‘whose staff require further motivation’^87^ A year later, Baily claimed that the laboratory quality control reports ‘clearly reflect the impact of the visits of the laboratory supervisor’ and, also as a result of Cobbald’s report, recording procedures had been simplified, and criteria for evaluating the slide been defined.^88^

Six years into the programme, Han and Richards stated that they were ‘very confident that, with the enthusiastic and active support of the Ministry and the Directorate of the Health Services, the Superintendent of the Anti-tuberculosis Campaign and his colleagues will have eventually succeeded in organising a sound rational national tuberculosis control programme’.^89^ However, their recommendations in this 1972 report indicated that the problems highlighted at the beginning of the project remained. It was noted, for example, that the number of sputum specimens collected was lower than expected and that this was ‘largely due to a lack of understanding’. What was needed in the training was

more emphasis on practical points, such as the need to be careful in selecting symptomatic patients, the need to make at least three consecutive sputum examinations, to be careful in the collection and preparation of proper sputum specimens, and to be prompt in packing and mailing slides to appointed microscopy centres so that they arrive quickly and in an undamaged condition.^90^

There was no lack of training in place. This included demonstration, in-service training, monthly meetings and seven working guidelines (manuals) for managers, peripheral health and medical personnel.^91^ This emphasis on the issue of training suggests that the practical difficulties of running the programme were being downplayed. This evident lack of resources was clearly a significant problem, but arguably as important were the willingness of doctors to adopt this methodology and the willingness of patients to use their local health agency when they had tuberculosis symptoms.

## 5 Problems of Institutional, Medical and Patient Culture

The WHO programme was not being transferred onto a blank page; recent scholarship has sounded the alert on assumptions that the objects of international health and development aid in colonial, post-colonial and independent states were passive recipients of these international initiatives and lacked agency of their own.^92^ The rationale behind the WHO tuberculosis control plan to extend tuberculosis control in an economically feasible manner to reach the rural areas of Sri Lanka made theoretical sense but, for it to work as it was designed to, it needed the cooperation of both the medical and technical professionals and of the general population. Although the WHO and other international health agencies had, in their early years, recognised the importance of understanding the cultural milieu of the recipients of aid, by the 1960s, there had been a shift away from these ideas of community development towards an emphasis on the transfer of technology.^93^ Ironically, although this model of tuberculosis control was deemed a community-orientated programme, it was, in reality, an example of a technical transfer that needed to override existing processes and patterns of behaviour in order to succeed. The two decades of the existing TCP had contributed to the growth of institutional, medical and patient cultures that would have to be supplanted by the new programme for it to be effective. It might have the support of those at the top of the health care infrastructure but how did those who had to work the system at the lower levels – the doctors, the technical staff and the patients – respond to it?

The programme had to be drafted onto the existing general health care infrastructure; this would entail changes to the administrative organisation and to the work of general and specialist medical and technical staff. Han and Richards raised the issue of the lack of integration at the top with the general health services at the divisional level in their quarterly report of March 1969 and reported that the divisional Superintendent of Health Services (SHS) and his staff ‘should participate more actively and directly with the project’. Only with this cooperation could a ‘properly functioning integrated tuberculosis control programme’ be achieved.^94^ A year later, they emphasised that it was essential for a successful integrated programme that the TB control officer had official status as a member of the SHS staff.^95^ Two years after this, in 1972, when the programme was being extended to the whole island, they were still making recommendations that changes be made to the administrative structure in order to ‘streamline communications’ and establish lines of authority. The existing administrative structure meant that the Superintendent of the Anti-tuberculosis Campaign had ‘no authority to issue any technical instruction directly to the periphery’ but had to go through the health services directorate and then to the SHS to issue them to the local health agency. Therefore, they suggested, the Superintendent of the Anti-tuberculosis Campaign should be designated Assistant Director of Health Services (TB) to give him official status within the health directorate.^96^ Continuity of staff at the general health agencies was also a problem. Under their conditions of service, MOs had the right to apply for transfers after two years in a post so that there was an annual rotation of about one third of all personnel. This applied, particularly, to health units in rural areas where MOs were reluctant to serve longer than they needed as these positions were regarded as stepping stones to better appointments. This meant that TB staff had to undertake crash training programmes with the newly appointed MOs on the integrated service every year and provide constant supervision.^97^

Suspicion of the new procedures from staff expected to implement the programme emerged from the outset. In the first months of the NWP pilot, Loven and Seneviratne reported that, on receiving a visit from their team to explain and demonstrate the new method, staff at the health agencies ‘started to realize that this project is not only a plan on paper but that it is becoming more and more a lively project’. Project leaders made surprise visits on any day at any time and ‘staff members in the various centres sometimes look quite surprised and “disturbed” at such activity’.^98^ Such attitudes might be expected, as Loven and Seneviratne commented, ‘it cannot be expected that since the inception of the project in May 1966 everything can be plain sailing’. Staff were going to ‘have to pull themselves up as regards output and quality of work’, they added and the DHS had already had to take ‘disciplinary action against some staffs who were caught for neglect of work’.^99^ In September 1967 Cobbald noted that it was ‘not easy for the staff at the centres, and was even more difficult for the patients, to accept the new approach immediately and completely’.^100^

In 1968 Dr G. Baily was given the task of conducting a study of the first full year of the sputum case-finding procedures in the pilot project from January to December 1967. His results did not indicate a high success rate for the favoured WHO method. Of the 3 934 173 patient visits at the ninety-two peripheral agencies of the province (that was an average of three visits per person), bacteriological examinations were made on 12 227 patients and fifty-one new cases (0.4 per cent) were confirmed. However, these fifty-one new cases were diagnosed by just thirty of these agencies. The remaining sixty-two agencies examined 7325 from 2339 592 patients but diagnosed no new cases. Hence, of the 177 newly detected cases in the province, 126 were diagnosed at the existing specialist clinics. Furthermore, the main chest clinic at Kurunegala was responsible for 108 of that 126; and, tellingly, of those 108 patients, 86 had by-passed their home agency and had come ‘of their own volition directly to the Clinic’.^101^ Baily’s study showed that the local agencies that did attract patients were only those located far from roads and means of transport and that people who lived ‘within reach of the public transport system tend to by-pass the peripheral agencies and travel to the clinic even from considerable distances’.^102^ Baily concluded that ‘the self-referral of such a high proportion of patients clearly implies a widespread public awareness not only of the symptoms of tuberculosis but of the health facilities to cure the patients’.^103^ It is also possible that the stigma associated with TB was an incentive for patients to avoid local agencies in order to keep their disease secret. A breakdown of two provinces in Han’s and Richards’ field report of July 1970 in Table 1 suggests that this bias in favour of the chest clinics continued.

**Table 1:** Case Finding by Sputum Testing in the North Western and Central Provinces. Source: Compiled from WHO Archives, Geneva, Project Files, Sri Lanka–75 1965-72 TUB 1, BCG/TBC, Dr Eung Soo Han and Dr A.H.D. Richards, Quarterly Field Report, 16 July 1970, Section III.1, 4.

Period	No of slides examined	Positives (microscopic)	Percentage
North Western Province			
April 1966/September 1969	31 580	549	1.7
January/March1970			
Chest clinics	252	57	22.6
Peripheral agencies	759	11	
Total	1 011	68	6.7
Central Province			
January/December 1969	2 954	178	6.0
January/June1970			
Chest Clinic	999	76	7.5
Peripheral Agencies	1 038	24	2.3
Total	2 037	100	4.9

In both of these examples, the peripheral agencies tested more patients but had fewer positives. Patient behaviour, as manifested in this preference for the existing and known specialist services, militated against the integrated programme as much as against lack of resources. It also merged with the resistance by the medical professionals to change long-adopted practices in favour of new methods.

The specialist chest clinics were intended to be a fundamental part of the new programme and were expected to adapt to the integrated programme, but it seemed that the MOs operating the specialist clinics, as well as other practitioners, were especially reluctant to adopt the new diagnostic method in the manner prescribed. Baily reported in 1968 that all patients attending the chest clinics for the first time were initially screened by X-ray, ‘and only those having pulmonary shadows are examined bacteriologically for acid fast bacilli’. This happened whether they had been referred by ‘peripheral agencies, general hospitals, or private practitioners’, or if they had come voluntarily ‘because of symptoms which they associate with tuberculosis’. Moreover, on the basis of a clinical examination of the X-ray, patients were placed on the ambulatory treatment regime even if their sputum tested negative.^104^ This diagnostic and treatment practice followed the existing method but went against all the guidelines of the new process. This did not go unnoticed by project leaders. Han and Richard’s Report of December 1969 praised the support they were receiving from those at the top of the administration but they foresaw ‘great operational difficulties in the Western Province which is scheduled to commence in June 1970 due to differences in opinion on the project particularly among senior TB officers’.^105^

In January 1971 at a one-day seminar attended by all MOs in the tuberculosis service, it was noted that ‘criticism was made of primary sputum case finding as against the conventional chest X-ray examination as a screening method’ and ‘the integrated scheme was also compared unfavourably with the patient-orientated chest clinic service programme’.^106^ Some insight into what these criticisms might encompass can be seen in the opening article of the April 1970 CNAPT monthly bulletin written by Dr Jayasuriya, respected chest surgeon, TB expert and CNAPT founder and chairman. It was an impassioned plea for professional knowledge and clinical skill over a laboratory test. There was no doubt, he argued, that a patient whose sputum tested positive was suffering advanced tuberculosis and would be highly infectious. However, he explained, ‘experienced clinicians know that for every case that is sputum positive there are perhaps 8 or even 10 cases of active tuberculosis that are sputum negative’.^107^ These patients could be diagnosed through a combination of radiology, Mantoux Test and the diagnostic skill of the clinician and treated before the disease advances and they become infectious. ‘Belittling’ this approach, he argued, would be ‘committing a serious offence’ against sufferers; and he stressed that the ‘transmitters of today were all sputum negative cases yesterday’. He claimed that in the discussions at the February 1970 WHO Seminar ‘the treatment of only the TB positive was condemned and that such a procedure was considered both morally and scientifically unsound’.^108^ The ‘needs and conditions for tuberculosis control’, he argued, ‘differ from country to country’ and if it was to be controlled in Sri Lanka ‘we must make fullest use of all the facilities for case finding and treatment that have been built up over these many years’.^109^ Jayasuriya’s comments reflect the tensions noted by both Amrith and Valier that emerged in the Madras Trials; ‘the potential disjunction between the commitment of the physician to cure each patient, and the demands on international public health officials to cure the greatest number at the lowest cost’.^110^ For Jayasuriya and his colleagues, treating the individual sufferer at the first signs of symptoms satisfied both their primary purpose of curing the individual but also the public health imperative of reducing infection in the community. Waiting for a positive sputum test before starting treatment did not make sense, especially when there was already a functioning control system in place. It seems that both patients and doctors were manifestly reluctant to adapt their behaviour to the new scheme and, without their cooperation, it would not work.

## 6 Concluding Remarks

In the period under discussion, there was a decline in the incidence of tuberculosis, as seen from the comparison in Table 2 of the two baseline sample studies of 1956 and 1970, conducted with the assistance of the WHO.

**Table 2:** Prevalence of Tuberculosis in 1956 and 1970.  Population 10 years and over: **Population 15 Years and over.  Source: WHO Archives, Geneva, Project Files, Sri Lanka–75 1965–72 TUB 1, BCG/TBC, Dr S. Grzybowski, Assignment Report on Tuberculosis Control, 25 June 1971, Annex 1, Table 8, 4.

	Survey population	No. of positives	Prevalence %	% of estimated prevalence of total population
1956	16 225*	26	1.602	1.146
1970	12 468**	16	1.285	0.743

An evaluation of Sri Lanka’s Tuberculosis Control Programme some twenty years later concluded that it had ‘had a positive impact on the epidemiological situation of tuberculosis’. There had been a shift to older age groups, making it more of a chronic disease, the notification of forty cases per 100 000 (all forms) in 1991 was attributed mostly to an efficient BCG vaccination programme, and it was noted that this was ‘low for a developing country’.^111^ However, the TCP programme that was evaluated in the report was not the envisaged community integrated programme of 1966. Although some branch chest clinics had closed in the early years of that programme, it was apparent that, despite the attempt at integration and some initial success, the WHO programme had not supplanted the existing specialist programme. In a 1986 review, Drs S.S.P. Gupta and S. Radhakrishna noted, that before 1969, 6300–6400 new cases of TB were detected and notified in the whole island, a peak being reached in 1975 of nearly 7300 when integration was at its height; since 1977, new TB case detection had slowed down but, more pertinently, the contribution of the general health agencies to new case detection had decreased. In 1977, 623 TB cases (sputum positive) had been detected by general health institutions but since then they had detected no more than 300–350 cases annually.^112^ Moreover, specialist chest clinics had not declined in importance; in 1986, twenty years after the integrated programme had started, there were now nineteen provincial chest clinics (up from seven) and three chest hospitals.^113^ Additionally, Dr Parrkali, in 1993, stated that diagnosis was confirmed by smear microscopy in less than fifty per cent of cases and he felt it necessary, as preceding advisers had, to stress the superiority of this method of diagnosis instead of ‘relying on X-ray findings’.^114^

Sri Lankans have suggested their own explanations for the failure of this technical transfer in their island. The resource problem was clearly salient for them. For example, the History section of the website of the National Programme for Tuberculosis Control and Chest Diseases in Sri Lanka states that the rapid rise in fuel prices at the start of the 1970s rendered supervisory visits virtually impossible and was a major factor in the failure of the integrated programme.^115^ In 1995, Dr C.G. Uragoda, a former head of Sri Lanka’s TCP and later Chair of the CNAPT, also emphasised that the WHO integrated programme was a success as long as the WHO poured resources into it but, as an ‘island-wide method, it became a failure’ and, moreover, that the main reason for its failure was ‘the lack of resources’. He too though emphasised the policy trajectory; he went on to say that ‘other countries laughed at us for dismantling a well-tried method’ for one ‘that was not tried nation-wide in any other country’. His pertinent advice in this address was that ‘great caution should therefore be exercised in abandoning a well-tried method of treatment in preference to a new method, whether on the advice of the WHO or any other agency’.^116^ Ultimately, in the case of tuberculosis control, Sri Lankans, whether medical professionals or sufferers of the disease, exercised their own agency. A community-orientated approach has to be just that and cannot be instigated from the top down or by outside experts.

In 1989, the Anti-Tuberculosis Campaign was renamed the Respiratory Disease Control Programme reflecting the improved tuberculosis situation and the coordination of TB control activities with other chest diseases. The National Programme for Tuberculosis Control and Chest Diseases (NPTCCD), the focal point for TB control today, still functions as a separate department within the Ministry of Health.^117^ Sri Lanka is not among the high burden countries for tuberculosis, but it remains a widespread problem and poses a continuing threat. This threat was highlighted in a March 2014 article in the Sri Lanka *Sunday Times.* The total number of detected cases in 2013, according to the Deputy Director of the NPTCCD, as quoted in the article, had been 9646, a case rate of 46 per 100 000. The estimated detection rate, according to WHO standards, ‘should be 66 per 100 000’, he stated; this meant that there were an estimated 4000 undetected sufferers. ‘Low income, lack of proper nutrition and low living conditions compound the issue’ but shortages of staff and microscopes, the difficulties of accessing health facilities and the fact that ‘TB has become a forgotten disease in health investigations’ were hampering efforts to deal with it.^118^ In the 2006–2015 Strategic Plan for Tuberculosis Control issued by the NPTCCD, notably, one of the Plan’s objectives was for the integration of the TB control services; to date, TB control services had been ‘implemented in a fairly vertical way with the district chest clinics as main centres of service delivery’.^119^ However, the Strategic Plan argued that ‘in the interest of long-term sustainability of the programme and to increase cost-efficiency, decentralization and integration in the general health care system are to be aimed for’.^120^ It remains to be seen how much more successful this planned integration is than that of forty years earlier, given the continuation of similar contextual factors.

## Footnotes

1.
WHO Expert Committee on Tuberculosis. Eighth Report, *WHO Technical Report Series, No. 290*, 1964, 17, para. 7, available online at www.who.int/iris/handle/10665/40606, accessed 2 June 2013. For an analysis of the genesis of this policy, see Sunil Amrith, ‘In Search of a “Magic Bullet” for Tuberculosis: South India and Beyond, 1955–65’, *Social History of Medicine*, 17:1 (2004), 113–30. For an overview of WHO policies for tuberculosis control, see M.C. Raviglione and A. Pio, ‘Evolution of WHO Policies for Tuberculosis Control, 1948–2001’, *The Lancet*, 359, 2 March (2002), 775–80.

2.
WHO *ibid.*, 3.

3.
For an overview of the global significance of tuberculosis, see Sue Bowden and Alex Sadler, ‘Review Article. Health Expectations and Health Achievements: Respiratory Tuberculosis in the Global Economy Between 1950 and 1980 – A developing Economy Perspective’, *Journal of International Development*, 26 (2014), 222–45.

4.
WHO *op. cit.* (note 1), 3. For an analysis of the significance of the new therapeutics in tuberculosis, see Helen Bynum, *Spitting Blood. The History of Tuberculosis* (Oxford: Oxford University Press, 2012), 189–229.

5.
The WHO Malaria Eradication Programme is the prime example of this belief in action. See, for example, Randall Packard, *The Making of a Tropical Disease. A Short History of Malaria* (Baltimore: Johns Hopkins University Press, 2007), 150–76.

6.
D. Mitchison and G. Davies, ‘The Chemotherapy of Tuberculosis: Past, Present and Future’, *International Journal of Tuberculosis and Lung Disease*, 16, 6 (2012), 724–32.10.5588/ijtld.12.0083PMC373608422613684

7.
Tuberculosis Chemotherapy Centre Madras, ‘A Concurrent Comparison of Home and Sanatorium Treatment of Pulmonary Tuberculosis in South India’, *Bulletin of the World Health Organization*, 21 (1959), 51–144; Helen Valier ‘At home in the colonies: the WHO-MRC trials at the Madras Chemotherapy Centre in the 1950s and 1960s’, in Fleurin Condrau and Michael Worboys (eds), *Tuberculosis Then and Now, Perspectives on the History of an Infectious Disease* (Montreal & Kingston: McGill-Queens’ University Press, 2010), 213–4; see Clare Leeming-Latham, ‘Unravelling the “Tangled Web”: Chemotherapy for Tuberculosis in Britain, 1940–70’, *Medical History*, 59, 2 (2015), 156–76 for an excellent analysis of the debates around the interaction of chemotherapy, surgery and bed rest as treatment options.

8.
Sunil Amrith, *Decolonising International Health. India and Southeast Asia, 1930–65* (Basingstoke: Palgrave Macmillan, 2006) 157.

9.
Mitchison and Davies, *op. cit.* (note 6), 2; Paul Farmer, Jim Yong Kim, Arthur Kleinman and Matthew Basilico (eds), *Reimagining Global Health. An Introduction* (Berkeley, CA: University of California Press, 2013), 235–9.

10.
Niels Brimnes, *Languished Hopes. Tuberculosis, the State and International Assistance in Twentieth Century India* (Hyderabad: Orient BlackSwan, 2016).

11.
See Sanjoy Bhattarcharya, *Expunging Variola. The Control and Eradication of Smallpox in India, 1947–77* (Hyderabad: Orient BlackSwan, 2006); Amrith, *op. cit.* (note 11); Condrau and Worboys (eds), *Tuberculosis Then and Now*; Niels Brimnes, *op. cit.* (note 10); Packard, *op. cit.* (note 5).

12.
See Margaret Jones, *Health Policy in Britain’s Model Colony. Ceylon (1900–48)* (Hyderabad: Orient Longman, 2004); Margaret Jones, *The Hospital System and Health Care, Sri Lanka 1815–1960* (Hyderabad: Orient BlackSwan, 2009); Kalinga Tudor Silva, *Decolonisation, Development and Disease. A Social History of Malaria in Sri Lanka*, (Hyderabad: Orient BlackSwan, 2014).

13.
Lauren Minsky, ‘Smallpox Prophylactic Practice in Colonial Punjab’, *Bulletin of the History of Medicine*, 83, 1, (2009), 164–90: 166: 165.10.1353/bhm.0.019819329846

14.
Amrith, *op. cit.* (note 11), 12: 15.

15.
Christian McMillen, *Discovering Tuberculosis. A Global History 1900 to the Present* (New Haven and London: Yale University Press, 2015), 64.

16.
National Archives, Kew (NA): CO 57/177, *Sessional Paper XIX, Report of the Tuberculosis Commission, 1910*.

17.
*Ibid.*.

18.
Sri Lanka National Archives (SLNA), *Administration Report of the Director of Medical and Sanitary Services, 1948, Tuberculosis*, C90. In the late 1940s, with the advent of DDT spraying, the annual incidence of malaria was rapidly falling; in the context of the time, this optimism did not seem to be misplaced. See: R.H. Gray, ‘The Decline of Mortality in Ceylon and the Demographic Effects of Malaria Control’, *Population)*, 28, 2 (1975), 205–29; Gordon Harrison, *Mosquitoes, Malaria and Man. A History of Hostilities Since 1880* (London: John Murray, 1978), 229–30; Jones, *Health Policy in Britain’s Model Colony*, 204–8.

19.
SLNA *Ibid.*, C90, C10.

20.
SLNA *Ibid.*, C91.

21.
SLNA, *Report of the Director of Medical Services, 1954, Tuberculosis*, B126.

22.
*Ibid*., para. 6 (3, 8), 4.

23.
See Bynum, *Spitting Blood*, 128–59, for an excellent survey of this institutional care. Collapse therapy refers to surgical procedures such as pneumothorax designed to rest the diseased lung. *The History of Tuberculosis* (Oxford: Oxford University Press, 2012).

24.
CNAPT *Report and Financial Statement 1953*, 6. Original copies of the CNAPT Reports and Bulletins were very kindly donated to the author by the present CNAPT Executive Secretary, Mr D.P. Fonseka.

25.
The CNAPT bulletins contain many examples of the effects of this stigma: wives deserted by husbands, daughters no longer deemed marriageable, a family who burned themselves alive because of the shame (CNAPT Report 1960).

26.
Dr J.H.F. Jayasuriya, OBE, FRCS, a prominent chest surgeon and who was Chairman of the Rotary Club, Colombo was the inspiration for its foundation and, for many years, he ran it from his home until it acquired its own building, in 1957, at fifty-one Sir Marcus Fernando Mawatha where it is still housed today. Dr B. Kaluarchchi, ‘CNAPT Serves the Nation, 60 Years, 17th June 1948–17th June 2008’, *The Island*, online, 17 June 2008, available at www.island.lk/2008/06/17/features2.html, accessed 10 November 2014.

27.
For BCG vaccination programmes in India at the same time, see Niels Brimnes, ‘Vikings against Tuberculosis: The International Tuberculosis Campaign in India, 1948–1951’, *Bulletin of the History of Medicine*, 81, 2 (2007), 407–30.10.1353/bhm.2007.002217844722

28.
SLNA, *Report of the Director of Medical and Sanitary Services 1950*, B137; CNAPT *Report and Financial Statement 1964*, ‘Address by Mr M.D.H. Jayawardena, Minister of Health’, 16–27, 17.

29.
SLNA, *Report of the Director of Medical and Sanitary Services, 1950*, para. 242, B137.

30.
SLNA, *Report of the Director of Medical and Sanitary Services, 1953*, para 45, B95: para 52, B96.

31.
*Report of the Director of Medical and Sanitary Services, 1956*, B14.

32.
*Ibid*., B14.

33.
NA DO/109, Sir Bennett Hance, *Sessional Paper XVI, Report on the Organisation of the Health Services of Ceylon*, para. 161.

34.
*Ibid*., paras 161: 65.

35.
*Ibid*., paras 163–5: 65–6.

36.
*Ibid*., para. 165, 66.

37.
WHO Archives, Geneva, Project Files, Sri Lanka–75 1965–72 TUB 1, BCG/TBC; A.PRO 529, *Plan of Operation for Tuberculosis Control in Ceylon*, signed by UNICEF, 2 September 1965, WHO, 3 September 1965 and the Government, 16 December 1965, 3; SLNA, *Report of the Director of Medical and Sanitary Services, 1956*, B157. Unfortunately I have not been able to locate a full copy of Dr James Deeny’s report. The WHO Archives have only a partial and largely illegible microfilmed copy.

38.
*Ibid*.

39.
‘CNAPT Pilot Project in Regional Organisation’, CNAPT *Report and Financial Statement, 1965*, 36–9, 37. The Jaffna project was a joint CNAPT, ITC and Government programme that relied on volunteers to ensure patient compliance with the bi-weekly drug regime; it ran alongside the WHO project.

40.
*Ibid*., 3.

41.
L. A. Simenov, *Better Health for Sri Lanka. Report on a Health Manpower Study, 1975*, 101–52; Jones, *The Hospital System and Health Care*, 311–16; Dr Susie Perera, ‘Primary health care reforms in Sri Lanka: aiming at preserving universal access to health’, in Alexander Medcalf, Sanjoy Bhattacharya, Hooman Momen, Monica Saavedra, Margaret Jones (eds), *Health for All. The Journey to Universal Health Coverage* (Hyderabad: Orient BlackSwan, 2015), 82–3.

42.
*Op. cit.* (note 38), 13–5.

43.
*International Work on Tuberculosis, 1949–1964*, 17, www.who.int.publications.international_work_tb_1949WHO. Accessed 25 June 2013.

44.
*Op. cit.* (note 38), 4.

45.
*Ibid*., 2.

46.
*WHO Expert Committee on Tuberculosis. Eighth Report*, 19, para. 7. 2.3.14199744

47.
*Op. cit.* (note 37); N. Cobbald, *Assignment Report on Laboratory Aspects of the Tuberculosis Control Programme, August–September 1967*, 5.

48.
Amrith, *op. cit.* (note 11), 129.

49.
See McMillen, *op. cit.* (note 15) for a specific example of this failure to learn the lessons in the case of a WHO TB control pilot project in Murang, Kenya, 156–8.

50.
Cobbald, *op. cit.* (note 46), 2.

51.
The National Tuberculosis Institute in Bangalore had been established in 1960 as a centre for planning, research and training in tuberculosis control and was considered the focus of expertise in the region. See Niels Brimnes, *op. cit.* (note 10).

52.
Cobbald, *op. cit.* (note 46), 1.

53.
*Ibid*., 2.

54.
*Ibid*., 3.

55.
*Ibid*., 4.

56.
*Ibid*., 4–5.

57.
H.E.R. Goonewardene, Executive Secretary, ‘From Unsupervised to Supervised Treatment in the Control of Tuberculosis’, *CNAPT Monthly Bulletin* VI, 1, January 1969, 6–11: 6.

58.
Cobbald, *op. cit.* (note 46), 4–5.

59.
See, for example, ‘Address by Dr J.H.F. Jayasuriya’, CNAPT, *Report and Financial Statement 1967*, 10.

60.
*Ibid*., 70–1.

61.
*Op. cit.* (note 37); Dr K.L. Hitze, RATB, *Tour Notes on Visit to Ceylon from 19–28 October 1966*, 3.

62.
Cobbald, *op. cit.* (note 46), 5.

63.
*Op. cit.* (note 37); Dr Eung Soo Han, WHO Project Leader and Dr A.M.D. Richards, Team Leader, *Quarterly Field Report, December 1969*, Paragraph 1.2, 1.

64.
*Op. cit.* (note 37); Dr Eung Soo Han, WHO Project Leader and Dr A. M. D. Richards, Team Leader, *Quarterly Field Report, 20 March 1970*, Remarks, 1.

65.
*Ibid*.

66.
*Ibid*., remarks, 2.

67.
*Op. cit.* (note 38), 13.

68.
*Op. cit.* (note 37); Dr K. L. Hitze, *op. cit.* (note 60), 1.

69.
*Ibid*, annex II, para. 2, 1. This lack of awareness of road conditions, which were very different from those of the developed world, crops up elsewhere in WHO projects. See, for example, M. Jones, ‘A “Textbook Pattern”? Malaria Control and Eradication in Jamaica, 1910–65’, *Medical History*, 57, 3 (2013), 397–41: 413–4.10.1017/mdh.2013.20PMC386594624069886

70.
*Op. cit.* (note 37); Dr F.J. Loven and Dr J.D. Seneviratne, ‘First Quarterly Report, 30 March 1968’.

71.
*Op. cit.* (note 37); Dr Eung Soo Han, WHO Project Leader and Dr A.H.D. Richards, Team Leader, *Second Quarterly Report, June 1969*, 3. Dr Richards had replaced Dr Seneviratne as project leader. Very few Europeans had worked in the Sri Lankan medical service since the 1920s and, uniquely, in the colonial context, a Sri Lankan had headed the service from 1936. It had always been predominantly an indigenous service (its medical college was founded in 1870). Tuberculosis seems to be one specialism where Europeans were still employed; another such tuberculosis specialist was Dr J. Wilson who also headed the TCP.

72.
*Op. cit.* (note 37); Dr Eung Soo Han and Dr A. H. D. Richards, *op. cit.* (note 62), 5.

73.
Eung Soo Han, *op. cit.* (note 70), para. 4, 3.

74.
*Op. cit.* (note 60), 2.

75.
Cobbald, *op. cit.* (note 46), 12.

76.
*Ibid*., 8.

77.
From 1966, for example, malaria began to re-emerge as a problem and, in 1968–69, once more reached epidemic proportions, increasing from under a 100 cases to half a million. Gordon Harrison, *Mosquitoes, Malaria and Man*, 254–5.

78.
*Op. cit.* (note 46), Annex 3, ‘Guidelines to Staff of Participating Health Agencies: Sputum Specimen Collection and Smear preparation’, para. 2, The Collection, 1.

79.
Eung Soo Han, *op. cit.* (note 63), Annex E, para. 7, 2.

80.
*Op. cit.* (note 77), paras 5.3, 9: 6.8, 12.

81.
Michael Worboys ‘Practice and the Science of Medicine in the Nineteenth Century’, 102, 1 *Isis* (2011), 109–15: 112.10.1086/65866021667779

82.
*Op. cit.* (note 37); Dr C.V.J. Baily and Mrs H. Patterson, *Assignment Report on Tuberculosis Control, 6 July–12 August 1968*, Para. 5, 13.

83.
Baily and Patterson, *ibid.*, para. 9.3, 21.

84.
*Op. cit.* (note 46), para. 5.4, 9.

85.
*Ibid*., para. 6.9, 15–16.

86.
*Ibid*., para. 6.2, 11.

87.
*Ibid*., para. 6.4, 11.

88.
Baily and Patterson, *op. cit.* (note 81), para. 5, 13.

89.
*Op. cit.* (note 37); Dr Eung Soo Han, WHO Project Leader and Dr A.H.D. Richards, Team Leader, *Assignment Report on Tuberculosis Control, 21 February 1972*, para. 6, 13.

90.
*Ibid*., para., 7.4.8, 15.

91.
A list of these guidelines can be found in Annex 3, Dr Eung Soo Han, *op. cit.* (note 88) and also in the curricula Dr Han devised for the training courses in *SEARO Memorandum, Performance Orientated Training Programme, Tuberculosis Control, 30 June 1971*.

92.
Bhattacharya, *Expunging Variola.*; Amrith, *op. cit.* (note 11); Paul Farmer, *Infections and Inequalities. The Modern Plagues* (Berkeley, CA, Los Angeles, CA, London: University of California Press, 2001).

93.
Kelley Lee, *The World Health Organization* (London, New York: Routledge, 2009), 5–9.

94.
Eung Soo Han, *op. cit.* (note 63), Annex E, para. 1, 1.

95.
*Ibid*., Section III, para. 4, 4.

96.
Eung Soo Han, *op. cit.* (note 88), para. 7, (1), 13.

97.
*Ibid*., para. 7, 2 (2), 14.

98.
Dr F.J. Loven and Dr J.D. Seneviratne, ‘Quarterly Field Report, 30 September 1966’, 1.

99.
*Ibid.*, 2.

100.
Cobbald, *op. cit.* (note 46), para. 3, 5.

101.
Baily and Patterson, *op. cit.* (note 81), para. 8.4, 19–20.

102.
*Ibid*., para. 4.4, 5.

103.
*Ibid*., para. 4.4, 5.

104.
*Ibid*., para. 4.5, 12.

105.
*Op. cit.* (note 37); Dr Eung Soo Han and Dr A.H.D. Richards, *Quarterly Field Report, 20 December 1970*, para. 6, III.1. 3.

106.
Eung Soo Han, *op. cit.* (note 88), para. 5.4, 11.

107.
Dr J.H.F. Jayasuriya, OBE, FRCS, Chairman of the CNAPT Council, ‘Extract from Address, WHO Seminar on “Control of Tuberculosis” in Ceylon’, CNAPT *Monthly Bulletin*, VII, 2, April 1970, 1–12: 5.

108.
*Ibid*., 9.

109.
*Ibid*., 6.

110.
Amrith, *op. cit.* (note 11), 159.

111.
WHO Archives, Geneva, Third Generation Files, TB/370/12/SL, Tuberculosis 1981–96, Dr Lissa M. Parkali, *Evaluation of Tuberculosis Control Activities in Sri Lanka, 10 May–10 June 1993*, para. 4.1, 8.

112.
*Ibid.*, Dr S.S.P. Gupta and Dr S. Radhakrishna, *Assignment Report, Assistance in the (1) Review of the Tuberculosis Control Programme, and (2) Preparation of a Plan on the Methodology of Prevalence Survey, Sri Lanka, 31 December 1985–30 January 1986*, para. 4, ‘Existing Situation’, 5.

113.
*Ibid*., para. 3.2, ‘Facilities’, 2.

114.
Parkali, *op. cit.* (note 111), para. 4.2 (3), 9.

115.
See the section ‘History’ on their website at www.healthlk/en/TB, accessed 7 July 2105.

116.
C.G. Uragoda, ‘Address by the Chairman, CNAPT’, CNAPT *Report and Financial Statement, 1995/6*, 5–7, 6. Chris Uragoda is the author of the seminal work *A History of Medicine in Sri Lanka* (Colombo: Sri Lanka Medical Association, 1987).

117.
National Programme for Tuberculosis Control and Chest Diseases, Ministry of Health, Sri Lanka, *Strategic Plan for the Tuberculosis Control, 2006–15*, available online at www.nptccdsrilanka/home, accessed 15 June 2014.

118.
Chathuri Dissanayake, *The Sunday Times*, 30 March 2014, ‘Sri Lanka fast developing into TB hub of Asia’ available at www.sundaytimes.lk/140330/news/sri-lanka-fast-developing-into-hub-of-asia-91034.html, accessed 13 May 2015. The article begins by highlighting the threat posed to Sri Lanka by the migration of TB infected labour from China and India into Sri Lanka to work on development projects.

119.
*Op. cit.* (note 116), 12.

120.
*Ibid.*, 1.

